# Tuberculosis Case Finding: Evaluation of a Paper Slip Method to Trace Contacts

**DOI:** 10.1371/journal.pone.0075757

**Published:** 2013-09-20

**Authors:** Judith Mwansa-Kambafwile, Kerrigan McCarthy, Varanna Gharbaharan, Francois W. D. Venter, Boitumelo Maitshotlo, Andrew Black

**Affiliations:** 1 TB/HIV Unit, Wits Reproductive Health and HIV Institute, Johannesburg, Gauteng Province, South Africa; 2 Department of Clinical Medicine, University of Witwatersrand, Johannesburg, Gauteng Province, South Africa; 3 Epidemiology Unit, The Aurum Institute, Johannesburg, Gauteng Province, South Africa; Wadsworth Center, United States of America

## Abstract

**Setting:**

South Africa has the third highest tuberculosis (TB) burden in the world. Intensified case finding, recommended by WHO, is one way to control TB.

**Objective:**

To evaluate the effectiveness and acceptability of a paper slip method for TB contact tracing.

**Method:**

TB patients were offered paper slips to give to their contacts, inviting them for TB screening. The number of contacts screened and the proportion diagnosed with TB was calculated. Contacts that returned to the clinic after receiving the slips were interviewed. A focus group discussion (FGD) with TB patients was held to determine their acceptability.

**Results:**

From 718 paper slips issued, a 26% TB contact tracing rate was found, with a 12% case detection rate. The majority (68%) of contacts were screened within 2 weeks of receiving the slip. Age and gender were not significantly associated with time to screening. 16% of the contacts screened did not reside with the TB patients. 98% of the contacts said the method was acceptable. FGD findings show that this method is acceptable and may prevent stigma associated with TB/HIV.

**Conclusion:**

This simple, inexpensive method yields high contact tracing and case detection rates and potentially would yield additional benefits outside households.

## Introduction

Over the last two decades, the rate of tuberculosis (TB) in South Africa has been on the increase [1]. The country is among the twenty two high TB burden countries in the world [2]. The WHO recommends the “3Is” for the control of TB: infection control, intensified case finding and isoniazid preventive therapy [3,4]. Case finding can be either passive or active. The former refers to screening people that present themselves to a healthcare facility for problems unrelated to TB whilst the latter involves actively going out into the community to look for cases. Active case finding has been shown to yield more TB cases than passive case finding [5].

Finding and treating TB cases early reduces transmission and morbidity. A study conducted in Kenya showed a substantial proportion (64%) of undiagnosed, untreated pulmonary TB cases in communities [6]. These patients continue to transmit infection further increasing the TB burden. Using passive case finding, the mean duration of TB smear positivity (point prevalence/incidence) before diagnosis among South African miners was found to be 0.17 years (95% CI: 0.00–0.48) for HIV-positive miners and 1.15 years (95% CI: 0.33–2.94) for HIV-negative miners [7]. This transmission period may be shortened with more active case-finding and rapid access to treatment. Active case finding by both door-to-door visits and community screening using a mobile van has shown to reduce the prevalence of culture positive disease from 6.5 per 1000 adults (95% CI: 5.1-8.3) to 3.7 per 1000 adults (95% CI: 2.6-5.0) [8].

Methods used for active case finding vary from household visits to community mobilisation with mobile vans, and the use of paper slips. Home visits restrict contact screening to household members, whereas TB transmission mainly occurs in the community [9]. Although household contacts may be at high risk of acquiring TB, the contribution of the total new TB caseload from family contacts is minimal [10]. Extending contact tracing beyond the household would substantially improve case finding.

Paper slips have traditionally been used in STI (sexually transmitted infections) programs to inform sexual partners of their risk of acquiring an STI. Wright and colleagues found that this method was acceptable to patients [11]. Using this method to trace contacts of TB patients is a potential answer to increasing case finding. Data from a study that used paper slips to trace contacts of TB patients showed that 22.3% of TB patients had at least one contact screened for TB [12]. Using this method, TB patients can distribute slips to contacts both inside and outside their homes.

The South African National Department of Health (NDoH) 2011 draft guidelines on conducting TB contact tracing state that Community Health Workers (CHW) will conduct home visits to trace contacts of TB patients. However, for patients residing outside the catchment area of the concerned facility, the paper slip method will be used [13].

Circumstances may make home visits impossible or inappropriate, namely: a high burden of TB cases at a facility, migrant or homeless persons, and situations of overcrowding and cohabitation of urban settlements with non-household members and persons living outside the facility area. The paper slip method is a targeted alternative to home visits in such circumstances.

This study aimed to evaluate the effectiveness of the paper slip method for TB contact tracing by determining the contact tracing rate, the case detection rate among contacts and the acceptability of this method to both TB patients and their contacts.

## Materials and Methods

### Ethics Statement

Informed written consent was obtained from all study participants. Ethics approval was granted by the University of Witwatersrand Human Research Ethics Committee (M110233).

This study formed part of a larger project (The Region F TB Blitz) that investigated point of care use of Xpert MTB/RIF (an automated molecular test for *mycobacterium tuberculosis* and resistance to rifampicin).

Data were collected from six Primary Healthcare (PHC) facilities in a densely populated inner city sub-district in Johannesburg, South Africa between November 2011 and April 2012.

The standard of care in terms of contact investigation at these PHC facilities was to tell TB patients to ask their contacts to screen for TB.

**Figure 1:**
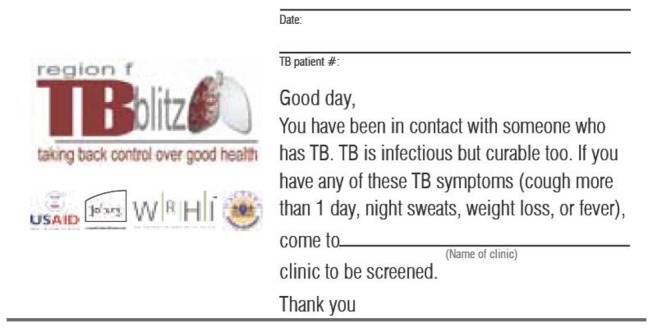
The paper slip with the message to invite contacts for TB screening.

Patients with pulmonary as well as extra pulmonary TB were offered small paper slips (90mm×50mm) to take to their contacts (the people they lived with and other people they spent time with during the day). These had no patient-identifiers and contained a message inviting contacts to test for TB (Figure 1). There was no limit to the number of slips that could be collected.

The contacts that presented for screening were invited to a short interview to determine their acceptability of the paper slip method. Questions were around how long it took for them to go for screening as well as their feelings about the process.

To determine the acceptability of the method by the TB patients, a focus group discussion (FGD) with TB patients that received paper slips was held at the end of the study. In order to ensure a representative group, selection was based on factors such as age, gender, number of paper slips taken, TB episode and number of months on TB treatment.

The TB contact tracing rate was defined as the proportion of contacts out of the total number of paper slips collected that went for TB screening because they received a paper slip. The TB case detection rate among contacts was defined as the proportion of contacts screened that were found to have TB.

We analysed the data using STATA 12 statistical software package [14]. We calculated the proportion of paper slips returned to the facilities, and the proportion of contacts diagnosed with TB. Using logistic regression, we calculated odds ratios (ORs) and 95% confidence intervals (CIs) to determine predictors of TB contacts going for screening. The analysis of qualitative data from the FGD was based on “grounded theory” [15].

## Results

From the total 718 paper slips issued, 189 (26%) were returned. The case detection rate among contacts screened was 12% (Table 1).

**Table 1:** Paper slips issued versus those returned.

*Clinic Name*	*# TB patients*	*# TB patients who got slips*	*# of slips issued*	*# returned*	*% returned*	*# TB positive*	*% TB*	*# Treatment started*
Clinic 1	119	60	108	33	31	4	12	4
Clinic 2	154	12	39	1	3	0	0	0
Clinic 3*	135	54	183	57	31	11	19	9
Clinic 4*	180	42	85	19	22	2	11	1
Clinic 5	91	30	58	18	31	0	0	0
Clinic 6*	218	66	245	61	25	5	8	2
Total	897	264	718	189	26	22	12	16

* Clinics that had TB contacts found to have rifampicin mono-resistant TB

Six contacts of TB patients were found to have rifampicin resistant TB on Xpert MTB/RIF and were referred to tertiary facilities for further management as per South African national TB guidelines.

During this period, there were 897 TB patients seen across the 6 facilities and 55% of them were smear or Xpert MTB/RIF positive (Table 2). Of these, 22 (2.4%) were TB contacts diagnosed after receiving a paper slip.

**Table 2:** Diagnostic methods for the TB patients during the 6 month period.

*Clinic Name*	*# TB patients*	*# smear + &/or Xpert MTB/RIF +*	*% smear + &/or Xpert MTB/RIF +*	# other diagnoses*
Clinic 1	119	85	71	34
Clinic 2	154	75	49	79
Clinic 3	135	113	84	22
Clinic 4	180	103	57	77
Clinic 5	91	31	34	60
Clinic 6	218	84	39	134
Total	897	491	55	406

^*^ Other diagnoses refers to diagnoses using TB diagnostic methods other than smear microscopy or Xpert MTB/RIF

### Acceptability of the method by TB contacts

Of the 189 paper slips returned, 141 contacts were interviewed, and of these 67% were female. The median age was 33 years (IQR: 26 to 40 years).

**Figure 2:**
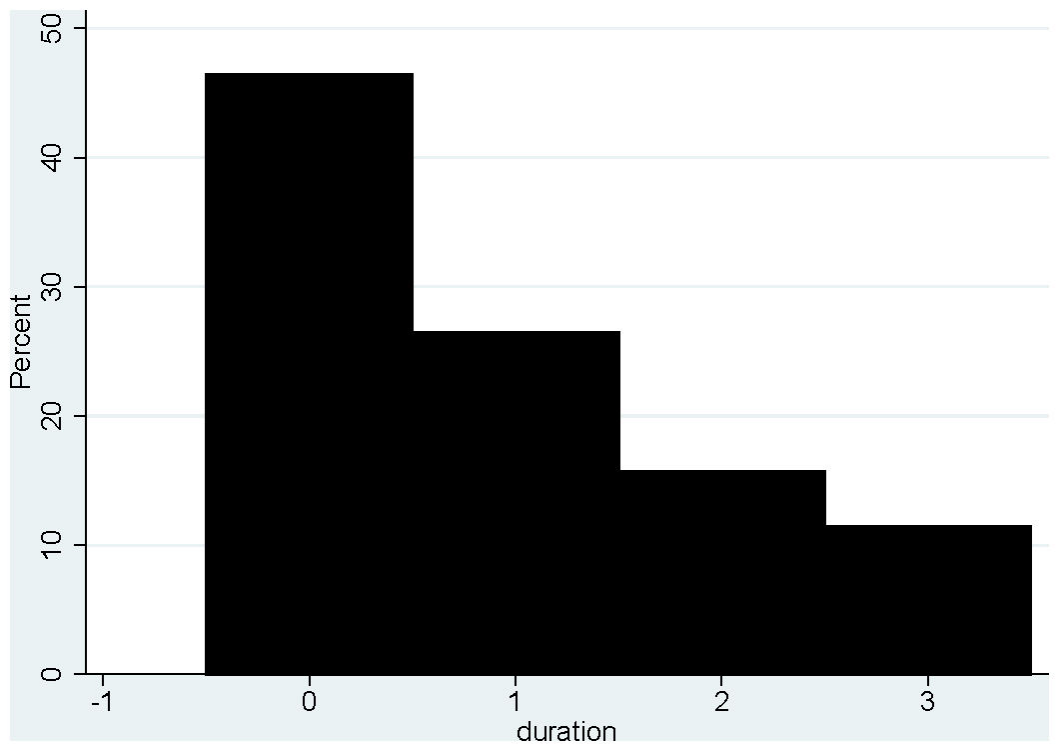
Time from receiving paper slip to TB screening. 0 = less than 1 week. 1 = 1 to 2 weeks. 2 = 2 to 4 weeks. 3 = more than 4 weeks.

The time that it took a TB contact to present for screening after receiving the message varied from less than 1 week to more than 4 weeks, with 68% presenting within 2 weeks of receiving the message (Figure 2). There were no significant differences between males and females as regards to time before TB screening (OR=0.78; 95% CI: 0.34 to 1.78). Age was not significantly associated with presenting early or late for TB screening (OR=1.0; 95% CI: 0.98 to 1.04).

Sixteen percent of the TB contacts did not live with the person who gave them the slip. Of these, the majority (60%) had daily contact with this person.

Overall, 11% of the TB contacts reported being “annoyed” when they initially received the paper slips.

Ninety eight percent of the contacts felt that paper slip notification is a good method to inform contacts. Of these, 10% reported being annoyed initially when they received the paper slip. However, they reported that their attitude softened when they went for screening.

### Acceptability of the method by TB patients

Of the 10 TB patients that were invited to the FGD, 6 consented. Their characteristics are shown in Table 3.

**Table 3:** Characteristics of TB patients in the Focus Group Discussion.

*Participant*	*Gender (female=F; male=M*)	*Age (years*)	*# of TB episode*	*# of months on TB treatment*
*Participant 1*	F	52	2	2
*Participant 2*	M	37	1	3
*Participant 3*	M	36	2	1
*Participant 4*	F	26	3	2
*Participant 5*	M	49	3	3
*Participant 6*	F	34	1	4

During the discussion, 4 participants recognized the paper slips immediately, and reported their emotions at the time they were asked to collect the slips. The initial feelings were generally centred on worry about stigma from the people around them.

One participant said…

*“I was shocked when I was asked to take slips. It was as if I was asked to announce to the whole world. The whole thing was too scary.”*

Another added…

*“I was hesitant to take the slips. I didn’t want people to know my illness. I even wanted to come and take my medicine from the clinic so that people won’t see I am on treatment.”*

All participants felt that the message on the slips was short and clear enough for their contacts to understand. One participant said …

*“I didn’t know what to say to my younger brother. It is his house, and I was worried that he would chase me out. So I decided not to tell him but to give him the slip so he reads for himself. He told me the message was clear and that he would go to the clinic. It was such a relief because I had mixed feelings about the whole thing.”*

Three of the participants felt it was easy to deliver the slips. These were all re-treatment cases. On the other hand, two other participants said that delivering the slips was like volunteering information about their illness and they were worried about people’s reactions.

One participant said …..

*“I threw away some slips after my housemates told me that I should not involve them and should deal with my illness alone. So *I* *didn* ’t even take slips to most other people I spend time with.”*

The other said …..

*“For me what shocked me the most was the reaction from my neighbours. They slammed doors on my kids and told their kids to stop playing with my kids and to stop coming to my house. That was very painful to witness.”*

## Discussion

Participants reported that men were less likely to visit healthcare facilities for a variety of reasons, including little time for clinic visits and the belief that a man visiting the clinic is not masculine. One woman said…….

*“Even though I told my partner and gave him the paper slip, he didn’t go to the clinic, saying he was too busy to go during the week, and clinics are closed on weekends.”*

To our knowledge, this is the first study that has looked at TB case detection rates among TB contacts traced by paper slips and the acceptability of this method to TB patients and their contacts. A South African study that used a similar method to ours to trace contacts of TB patients found a contact tracing rate of 22.3% [12]. However, this study did not ascertain how many of the contacts traced had TB and if the method was acceptable. Our results show that paper slips, which are easy and inexpensive to administer, may have a role in TB contact tracing. This method has the potential to increase case finding, prevent TB transmission, reduce TB incidence and generally improve success of TB programs.

TB contact tracing through home visits requires adequate resources and is labour intensive. Home visits may not play a significant role in reducing TB in communities since studies have shown that family contacts contribute minimally to the total TB caseload in the communities [9,10]. In our study, 16% of the slips were distributed beyond the household, and it would seem that this could be expanded with patient education, to arenas such as the workplace, churches and other congregation points, significantly improving the community penetration beyond the household. Although no cost effectiveness analysis of the paper slips method for TB contact tracing has been done, the method does not require additional staff or incur major costs beyond the printing of the slips.

Home visits to trace contacts may be unpopular due to TB patients’ discomfort with being visited at their homes. The advantage of the paper slip method in this regard is that the slip can be anonymous (if not handed to a person directly).

Although Kwazulu Natal province of South Africa has the highest TB prevalence in the country [16], we found a higher contact tracing rate (26% versus 22.3%) than was reported in a study conducted in that area [12]. This may be because inner city Johannesburg, where our study was done, is characterized by overcrowding in the households, pubs and other social/recreational facilities and this enables TB patients to have many contacts.

The 12% case detection among contacts that we found is more than twice (4.5%) that reported by a systematic review of household contact investigation [17]. The types of dwelling in our study area facilitate easy TB transmission [18]. The 3% rifampicin resistant TB that we found is in line with the fact that this is on the increase as was found by Mukinda and colleagues in a study they conducted in an area in South Africa with similar TB prevalence as our study setting [19].

### Acceptability of the method

Analysis of the time period it took for contacts to get screened for TB showed that the majority of them (68%) presented to the clinics within 2 weeks of receiving the paper slip. Women appeared to present themselves to the clinic more quickly than men. Although statistically insignificant (OR=0.78; 95% CI: 0.34 to 1.78), this finding in addition to more women being interviewed, is in line with the fact that there is more health seeking behaviour among women [20]. However, this is not easy to validate because the proportions of men and women who actually received the slips cannot be ascertained in our data. It is possible that there were more women than men who had received the slips hence more female contacts presenting for screening.

HIV and TB stigma is a problem in the community. Stigma can lead to discrimination of the patients by their friends and relatives and possibly resulting in reduced health seeking behavior among the patients [21]. The participants of the FGD felt that the paper slip method is an ideal way to inform their contacts.

### Limitations and Recommendations

Only symptomatic contacts were invited for TB screening. The number of TB cases could have been higher if asymptomatic contacts were also screened since a significant proportion of TB cases can be asymptomatic [22].

We could not ascertain the number of contacts attributed to an individual TB case because index TB case reference numbers were not consistently filled in on the slips.

Although cost effectiveness of this method was not evaluated, evidence suggests that such an intervention in an area with a high TB burden, is implementable and cheap through reducing the total TB caseload over time [23].

The majority of the paper slips were distributed to other household members; 16% of the slips returned were from contacts outside of the home. This is an important factor, as the majority of TB is transmitted outside of the household [9]. This suggests that with more education and explanation as the slips are being issued, this percentage could go higher. The slips should be used for workplace, church, sporting events and other venues in addition to household members.

We conducted only one FGD. A series of FGDs would have been ideal in order for us to make meaningful conclusions as regards to rejecting or supporting the information collected.

## Conclusion

Tracing contacts of TB patients using the paper slips method is a simple and acceptable way to ensure that contacts are screened for TB, and may be a useful adjunct to home visit programs, and a replacement in situations where home visits are not possible. It has the potential to improve TB case detection significantly, prevent TB transmission, reduce TB incidence and generally improve success of TB programs.
